# Interfacial Atom‐Substitution Engineered Transition‐Metal Hydroxide Nanofibers with High‐Valence Fe for Efficient Electrochemical Water Oxidation

**DOI:** 10.1002/anie.202115331

**Published:** 2022-01-28

**Authors:** Ben Zhang, Zihe Wu, Wenjie Shao, Yun Gao, Weiwen Wang, Tian Ma, Lang Ma, Shuang Li, Chong Cheng, Changsheng Zhao

**Affiliations:** ^1^ College of Polymer Science and Engineering State Key Laboratory of Polymer Materials Engineering Sichuan University Chengdu 610065 China; ^2^ College of Biomass Science and Engineering Sichuan University Chengdu 610065 China; ^3^ Department of Ultrasound West China Hospital Sichuan University Chengdu 610065 China; ^4^ Functional Materials Department of Chemistry Technische Universität Berlin Hardenbergstraße 40 10623 Berlin Germany

**Keywords:** Electrocatalysts, Hierarchical nanostructures, Metal hydroxides, Oxygen evolution reaction, Water splitting

## Abstract

Developing low‐cost electrocatalysts for efficient and robust oxygen evolution reaction (OER) is the key for scalable water electrolysis, for instance, NiFe‐based materials. Decorating NiFe catalysts with other transition metals offers a new path to boost their catalytic activities but often suffers from the low controllability of the electronic structures of the NiFe catalytic centers. Here, we report an interfacial atom‐substitution strategy to synthesize an electrocatalytic oxygen‐evolving NiFeV nanofiber to boost the activity of NiFe centers. The electronic structure analyses suggest that the NiFeV nanofiber exhibits abundant high‐valence Fe via a charge transfer from Fe to V. The NiFeV nanofiber supported on a carbon cloth shows a low overpotential of 181 mV at 10 mA cm^−2^, along with long‐term stability (>20 h) at 100 mA cm^−2^. The reported substitutional growth strategy offers an effective and new pathway for the design of efficient and durable non‐noble metal‐based OER catalysts.

## Graphical abstract

An electrocatalytic oxygen‐evolving NiFeV nanofiber was synthesized by an interfacial atom‐substitution strategy to boost the activity of the NiFe centers. The electronic structure analyses suggest that the NiFeV nanofiber exhibits abundant high‐valence Fe via a charge transfer from Fe to V. The reported substitutional growth strategy offers an effective and new pathway for the design of efficient and durable non‐noble metal‐based oxygen evolution reaction (OER) catalysts.

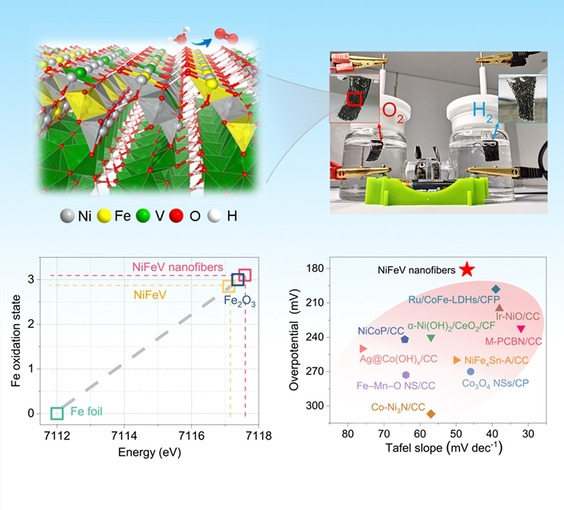

## Introduction

Electrocatalytic water splitting is a promising process for the preparation of molecular H_2_, which is conducive to meeting urgent requirements of becoming “carbon neutral” and overcoming the challenges of global warming.^[1]^ The current impediment for water electrolysis mainly stems from the four‐electron coupled process in the oxygen evolution reaction (OER) of the electrocatalysts at the anode, which suffers from the low efficiency and high kinetic barrier.^[2]^ Designing high‐performance electrocatalysts could effectively reduce the overpotential and overcome the sluggish kinetics of OER. To date, Ir‐ and Ru‐based catalysts present outstanding OER activity,^[3]^ but the widespread application of these noble metals is hampered by their extremely rare natural reserves.^[4]^ Therefore, it is important to develop low‐cost electrocatalysts from non‐noble metals for efficient and robust OER electrocatalysis.

Recently, numerous efforts have been made to develop highly efficient and earth‐abundant metal‐based OER catalysts, such as transition metal nitrides,^[5]^ oxides,^[6]^ phosphides,^[7]^ sulfides,^[8]^ and hydroxides.^[9]^ Among them, the NiFe‐based materials, particularly NiFe hydroxides, were explored as the most efficient OER electrocatalysts in alkaline media.[^4b^, ^10^] To further improve the OER performance of NiFe hydroxides, several effective strategies have been investigated, for instance, combining with conductive materials to improve electron transfer capacity,^[11]^ optimizing the ratio of Ni to Fe,^[12]^ manufacturing defects to modulate the electronic structures,^[13]^ and exposing more active sites by tailoring thickness.^[14]^ Apart from these methods, introducing a third transition metal (e.g., Co,^[15]^ Cr,^[9a]^ Al,^[16]^ and V^[17]^) has been considered as an effective strategy, which can effectively raise the Fe^3+^ ratio to enhance the electrocatalytic kinetics and lower the overpotential, the V dopants, in particular, have been recognized as one of the most efficient metals for boosting the OER electrocatalytic activity of NiFe hydroxides in alkaline solution.^[17a]^ However, the conventional one‐pot synthesis of V‐doped NiFe electrocatalysts may lead to low controllability of the electronic structures of NiFe centers and limited promotion on the Fe valence state. Therefore, it is pivotal to find a new path for the design of functionalized NiFe hydroxides with abundant high‐valence Fe via controllable V substitution.

Herein, we report an interfacial atom‐substitution strategy to synthesize an electrocatalytic oxygen‐evolving NiFeV nanofiber to boost the activity of NiFe hydroxides. We first fabricated V_3_O_7_ nanofibers as templates and V sources to achieve the controllable V substitution in the NiFeV nanofibers during the growth of NiFe hydroxides. The electronic structure analyses suggest that the NiFeV nanofiber exhibits abundant high‐valence Fe via a charge transfer from Fe to V. The electrocatalytic tests show that the NiFeV nanofiber displays a low overpotential of 263 mV at 10 mA cm^−2^. Meanwhile, the NiFeV nanofiber supported on a carbon cloth (CC) shows an ultralow overpotential of 181 mV to attain 10 mA cm^−2^, along with long‐term stability (>20 h) at a high current density of 100 mA cm^−2^. Especially, the constructed alkaline water electrolyzer using commercial Pt/C/CC and NiFeV nanofibers/CC electrodes deliver a current density of 10 mA cm^−2^ at a low cell voltage of 1.47 V, as well as favorable durability, therefore holding great potential for practical application. It is believed that the interfacial atom‐substitution strategy offers an effective strategy to optimize the OER performance of NiFe catalysts and enhance their activity and stability for water electrolysis, thus opening a general path for the rational design of highly efficient and durable non‐noble metal‐based OER catalysts.

## Results and Discussion

**Figure 1:**
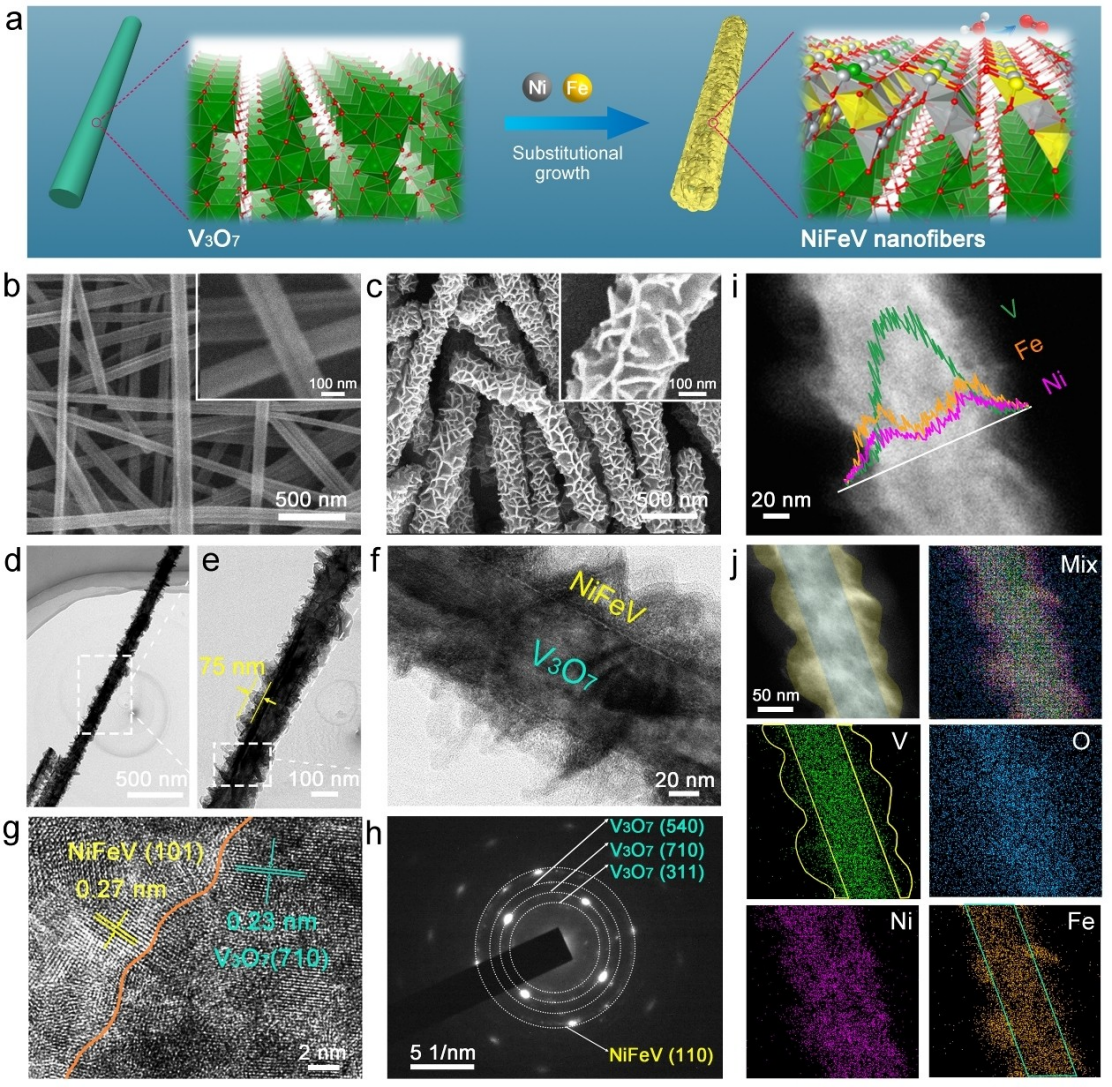
a) Schematic image for the synthesis of NiFeV nanofibers. SEM images of b) V_3_O_7_ nanofibers and c) NiFeV nanofibers, where the insets are the corresponding high‐resolution images. d–f) TEM images of NiFeV nanofibers at different magnifications. g) HRTEM image, h) SAED pattern, i) EDS line scan, and j) elemental mapping of NiFeV nanofibers.

As shown in Figure 1a, the NiFeV nanofiber is synthesized by using a V_3_O_7_ nanofiber as template and V source to achieve the controllable V substitution during the growth of NiFe hydroxides via a solvothermal reaction (see the experimental section in the Supporting Information).

Figure 1b shows that V_3_O_7_ nanofibers have a uniform diameter of about 120 nm, as indicated by the scanning electron microscopy (SEM) images. After substitutional growth of NiFeV hydroxide, a hierarchical nanofiber structure with rougher surfaces was obtained (Figure 1c and its inset). Transmission electron microscopy (TEM) was conducted to characterize the microstructure of NiFeV nanofibers further, showing that NiFeV nanosheets vertically grow on the V_3_O_7_, which forms a nanostructure with abundant edges and defects (Figure 1d–f). The diameter of the NiFeV nanofiber is approximately 270 nm, in which the NiFeV shell is about 75 nm. As revealed in the high‐resolution TEM (HRTEM) image (Figure 1g), the interface between the V_3_O_7_ (lattice spacing: 0.23 nm corresponding to (710) plane, cyan) and NiFeV (lattice spacing: 0.27 nm corresponding to (101) plane, yellow) can be clearly observed, indicating the substitutional growth of NiFeV from V_3_O_7_ nanofiber template. The selected area electron diffraction (SAED) pattern (Figure 1h) further validates the presence of NiFeV and V_3_O_7_ phases. The energy‐dispersive X‐ray spectroscopy (EDS) line scan results (Figure 1i) and EDS mapping analysis (Figure 1j) were applied to detect the spatial distribution for different elements in the NiFeV nanofiber. It is shown that the Ni and Fe are homogeneously distributed throughout the nanofiber, while O species are more concentrated in the core. It is noteworthy that V species are evenly doped into the shell (NiFeV hydroxides) in addition to being concentrated in the core (V_3_O_7_). The above results demonstrate that the NiFeV nanofiber consists of a core (V_3_O_7_)‐shell (NiFeV) heterostructure.

Thus, we predict that the V_3_O_7_ nanofiber templates can gradually release V atoms to coprecipitate with Fe, Ni, and hydroxyl ions to form NiFeV hydroxide nanosheets along the nanofiber during the solvothermal process. In order to reveal the important role of reaction time in regulating the growth and performance of NiFeV nanofibers, we have evaluated the materials obtained from 1 h to 24 h by monitoring their morphologies (Figure S1, Supporting Information), electronic structures (Figure S2 and S3, Supporting Information), and electrocatalytic activities on OER (Figure S4, Supporting Information). Under steady control of the reaction time, well‐defined oxygen‐evolving NiFeV nanofibers can finally be obtained, and 15 h is the optimal synthetic condition for efficient OER. Additionally, a series of catalysts were fabricated by directly regulating the proportion of Ni over the Fe; the optimum performance was found when the feed ratio of Ni to Fe was 3 to 1 (Figure S5, Supporting Information). It is noteworthy that when iron(III) nitrate nonahydrate replaces iron(II) sulfate heptahydrate as the iron source, the fiber structure of the V_3_O_7_ is destroyed, and the catalytic performance of OER decreased (Figure S6, Supporting Information). For comparison, pure NiFeV and NiFe nanosheets (Figure S7 and S8, Supporting Information) were also prepared by a similar solvothermal reaction.

**Figure 2:**
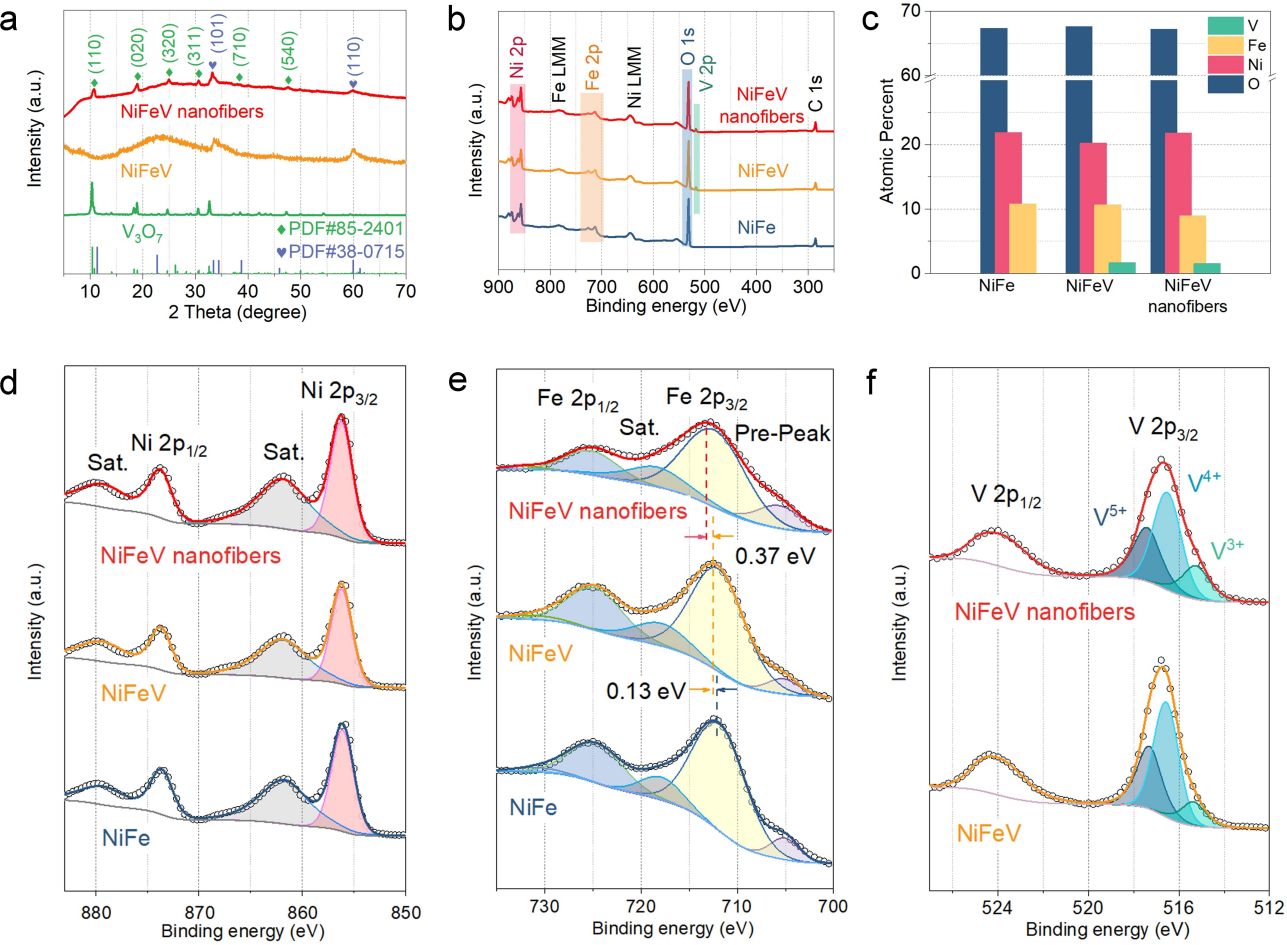
a) XRD patterns and b) XPS survey scans of the NiFe, NiFeV, and NiFeV nanofibers. c) Atom ratios of different catalysts. XPS spectra of d) Ni 2*p*, e) Fe 2*p*, and f) V 2*p* of different catalysts.

The X‐ray diffraction (XRD) pattern of the hierarchical NiFeV nanofibers is presented in Figure 2a, together with V_3_O_7_ and NiFeV as references, which confirms the successful synthesis of heterostructures after corresponding solvothermal treatment. The XRD spectrum of NiFeV nanofibers shows a series of peaks located a 2*θ*=10.8°, 18.9°, 24.7°, 30.7°, 38.4°, and 47.2°, which can be assigned to the (110), (020), (320), (311), (710), and (540) planes of V_3_O_7_, respectively (PDF #85‐2401), and the peaks at 2*θ*=33.5° and 60.0° match very well with the (101) and (110) planes of α‐Ni(OH)_2_, respectively (PDF #38‐0715). The prepared NiFeV and NiFe exhibit poor crystallinity and are isostructural to α‐Ni(OH)_2_ (Figure 2a and Figure S9, Supporting Information). To further investigate the compositions and electronic structures of NiFeV nanofibers, X‐ray photoelectron spectroscopy (XPS) was undertaken. Figure 2b shows the XPS survey spectra, indicating the presence of Ni, Fe, and O in the NiFe, and Ni, Fe, V, and O in the NiFeV and NiFeV nanofibers, which is consistent with the EDS results (Figure S10, Supporting Information). The surface composition analysis by XPS, Figure 2c, suggests the NiFeV nanofibers have a similar atom ratio to the pure NiFeV hydroxides (Figure 2c and Table S1, Supporting Information). For NiFeV nanofibers, the Ni 2*p* spectrum of XPS survey scans (Figure 2d) exhibits an apparent 2*p*_3/2_ peak at 856.2 eV along with a shakeup satellite at 861.8 eV, which are characteristic peaks of divalent Ni.^[18]^ The Ni 2*p*_3/2_ peaks of the NiFeV and NiFeV nanofibers show a very slight positive shift in binding energy compared to pure NiFe, with the shift value of 0.13 eV for NiFeV nanofibers and 0.07 eV for NiFeV. The fairly broad Fe 2*p*_3/2_ peak (712.4 eV) can be attributed to Fe in the 3^+^ oxidation state (Figure 2e), which is accompanied by a tiny peak at 705.6 and 718.3 eV corresponding to the pre‐peak and satellite peak, respectively.[^11^, ^19^] Particularly, the peak at 711.94 eV from the Fe 2*p*_3/2_ orbital in the NiFe was increased to 712.07 eV in the NiFeV (Δ*E*=0.13 eV) and 712.44 eV in the NiFeV nanofibers (Δ*E*=0.50 eV) (Figure 2e). The V 2*p* region consists of V 2*p*_3/2_ and V 2*p*_1/2_ (Figure 2f), and the peaks at 517.4, 516.5, and 515.2 eV can be indexed to V^5+^, V^4+^, and V^3+^, respectively.[^17a^, ^20^] It's worth noting that the proportion of low oxidation state vanadium (V^3+^) in NiFeV nanofibers is higher than that in NiFeV (Figure S11, Supporting Information). The high‐resolution XPS analyses indicate that the interfacial atom‐substitution strategy results in more electron transfer to the V in NiFeV nanofiber than that in NiFeV. This phenomenon can be attributed to the stronger interaction between NiFe and V, which further supports the atomic doping of V into NiFe hydroxide. In the O 1s region, a peak that occurs at 531.4 eV indicates oxygen ions in metal hydroxides (Figure S12, Supporting Information).^[21]^

The increasing binding energy of Fe in NiFeV nanofibers suggests a higher valence state, which is advantageous to the OER performance.^[22]^ These shifting phenomenons are likely caused by strong electronic interaction between V_3_O_7_ and NiFeV, which can change the local electron distribution, thus regulating the adsorption energies of oxygen intermediate species and tuning the catalytic performance.[^11^, ^23^]

**Figure 3:**
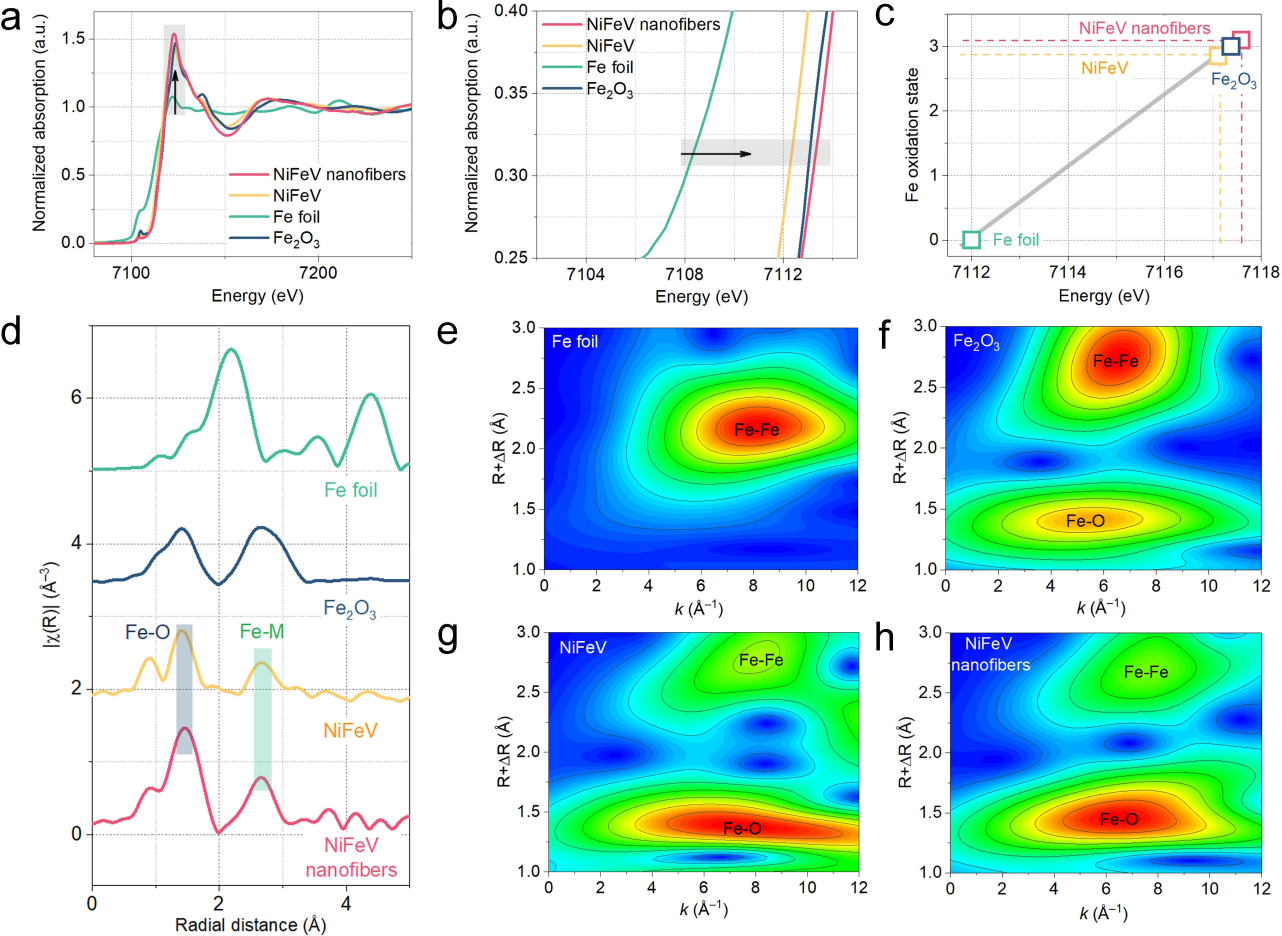
a, b) Fe K‐edge XANES spectra, c) Fe oxidation state, and d) Fourier‐transformed k^3^‐weighted EXAFS spectra of NiFeV, NiFeV nanofibers, and standard samples. e–h) WT analysis at the Fe K edge of different samples.

Furthermore, X‐ray absorption structure (XAS) spectroscopy measurements at the Fe K‐edges were carried out to compare the electronic properties of NiFeV nanofibers and NiFeV as a function of controllable V substitution in the NiFeV nanofiber. The normalized X‐ray absorption near edge structure (XANES) spectra for the Fe K‐edges of NiFeV nanofibers, NiFeV, and standard samples are shown in Figure 3a, b. Compared with NiFeV and standard reference samples, the absorption edge of NiFeV nanofibers displays increased white‐line intensity and obvious positive shift, manifesting the highest average Fe valence state.^[11]^ We have utilized the absorption threshold value (*E*_0_) to calculate the valance states of the Fe atoms (Figure 3c), the NiFeV nanofibers show the highest Fe valance states (>3^+^) than the NiFeV (<3^+^) and Fe_2_O_3_ (≈3^+^), which is in good accordance with XPS data.^[24]^

The extended X‐ray absorption fine structure (EXAFS) spectra of the NiFeV nanofibers and reference samples provide further structural information (Figure 3d). The peak at about 1.5 Å is ascribed to Fe−O coordination, thus validating an oxidized state of Fe species.^[25]^ The relatively weak peak at about 2.7 Å refers to Fe−M coordination, where M represents Fe, Ni, or V atoms.[^25a^, ^26^] Similar results are confirmed in the wavelet transform (WT) analyses of the Fe K‐edge data (Figure 3e–h).

**Figure 4:**
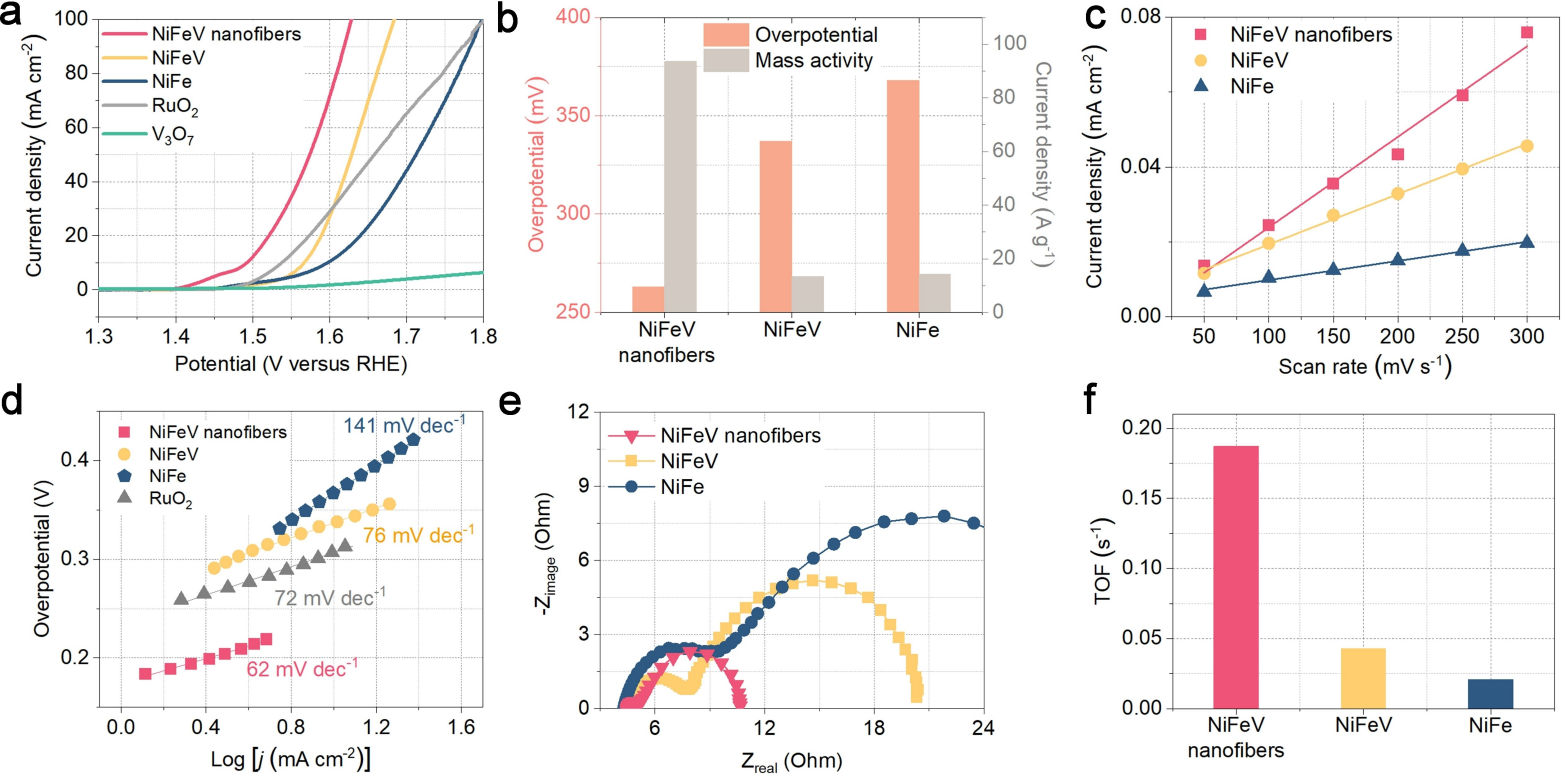
a) OER polarization curves of different catalysts in 1 M KOH. b) Comparison of the overpotentials at 10 mA cm^−2^ and normalized current densities based on mass at 1.53 V versus RHE. c) *C*_dl_ plots inferred from CV curves, d) Tafel plots, e) Nyquist plots, and f) TOF (at an overpotential of 350 mV) for NiFeV nanofibers, NiFeV, and NiFe electrocatalysts.

The OER performances of various catalysts were measured in a three‐electrode system with catalyst‐modified rotating disk electrodes (RDE). Figure 4a shows the linear sweep voltammetry (LSV) of as‐prepared samples in Ar‐saturated KOH solution, and the NiFeV nanofibers exhibit an overpotential of 263 mV at 10 mA cm^−2^, which is much lower than the NiFeV hydroxides (337 mV), commercial RuO_2_ (308 mV), and previously reported catalysts (Figure 4b and Table S2, Supporting Information). It can be seen that the bare V_3_O_7_ has almost no OER activity, which demonstrates that the OER activities are contributed by the in‐situ formed NiFeV nanosheets via interfacial atom‐substitution strategy. To evaluate the intrinsic OER activity, the current densities were normalized by electrochemically active surface area (ECSA) and the loading mass of the catalysts at the overpotential of 300 mV (Figure 4b and Table S3, Supporting Information).[^3a^, ^17a^] As observed, the NiFeV nanofibers possess the highest specific activity (11.11 mA cm^−2^) and mass activity (93.6 A g^−1^) (Table S3, Supporting Information). The calculated electrochemical double‐layer capacitance (*C*_dl_) and ECSA exhibit a similar trend to the OER activity (Figure 4c, Figure S13, Table S3, Supporting Information). The ECSA value of NiFeV nanofibers is larger than that of NiFeV and NiFe, indicating that the NiFeV nanofibers contain more oxygen‐evolving sites. The vertical geometry and fiber structure not only increase the Brunauer–Emmett–Teller (BET) surface area and the electrochemical active surface area, which accounts for the increased catalytic surface sites of NiFeV nanofibers, and thus contributing to their OER performance (Figure S14, Supporting Information).

As shown in Figure 4d, the Tafel slope of the alkaline OER on NiFeV nanofibers is 62 mV per decade (mV dec^−1^), which is smaller than NiFe (141 mV dec^−1^), NiFeV (76 mV dec^−1^), and RuO_2_ (72 mV dec^−1^). Accordingly, the NiFeV nanofibers proceed a faster oxygen evolution kinetics. Moreover, electrochemical impedance spectroscopy (EIS) has been performed to examine the electron‐transfer kinetics during the electrocatalytic process (Figure 4e). The semicircles in the high‐frequency zone are related to charge‐transfer resistance within electrodes (R_H_), while the lower frequency region semicircles are attributed to charge‐transfer resistances at the electrode‐electrolyte interface (R_L_).[^17a^, ^22a^, ^27^] Both semicircle diameters apparently decrease in the order of NiFe, NiFeV, and NiFeV nanofibers, indicating the NiFeV nanofiber has the fastest electron‐transfer during OER. The turnover frequency (TOF) value of the NiFeV nanofibers has also been calculated, which is 8.9 and 4.3 times higher than that of the bare NiFe and NiFeV, respectively (Figure 4f). Therefore, it is conceived that the NiFeV nanofibers manifest the highest intrinsic OER activity due to the heterostructure and abundant high‐valence Fe.

**Figure 5:**
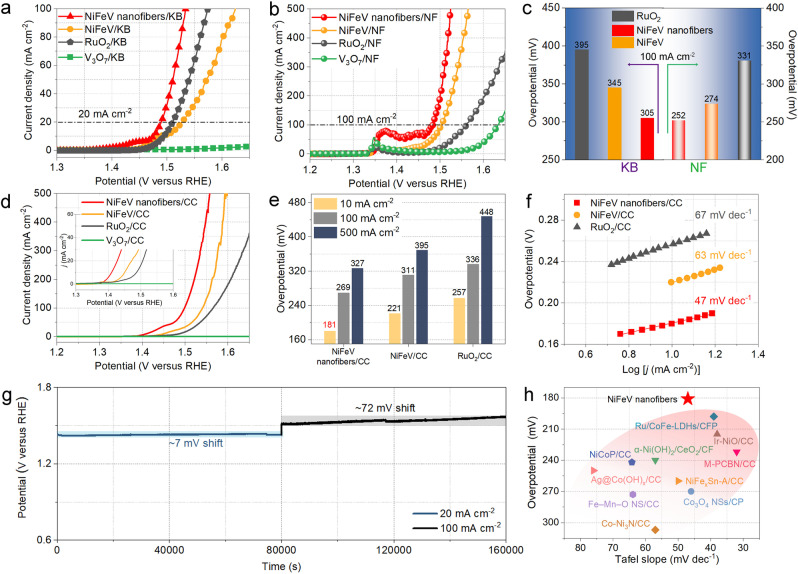
a) LSV curves of KB modified OER catalysts tested by RDE. b) LSV curves of different catalysts loaded on nickel foam. c) Extracted overpotentials from different catalysts with KB modification or NF loading at 100 mA cm^−2^. d) LSV curves, e) the overpotentials at 10, 100, and 500 mA cm^−2^, and f) the corresponding Tafel plots of different catalysts loaded on CC. g) Chronopotentiometric curves obtained from the NiFeV nanofibers loaded on CC at the constant current densities of 20 and 100 mA cm^−2^. h) Comparison of overpotentials at 10 mA cm^−2^ and Tafel slope of NiFeV nanofibers/CC with those reported state‐of‐the‐art OER catalysts on conductive supports (CC: carbon cloth; CP: carbon paper; CF: carbon foam; CFP: carbon fiber paper).

As known, electrical conductivity plays a crucial role in achieving superior performance toward OER.[^2a^, ^4b^, ^11^] First, the catalysts were modified by Ketjen Black (KB) at a weight ratio 9 : 1; the modified catalysts show superior OER activity to the original catalysts (Figure S15, Supporting Information). The KB modified NiFeV nanofibers (NiFeV nanofibers/KB) only require an overpotential of 259 mV to drive a current density of 20 mA cm^−2^ (Figure 5a). Moreover, we found that increasing the loading amount will also be beneficial to further boost the OER catalytic activity of the electrocatalysts (Figure S16, Supporting Information).^[28]^

Besides KB, we also investigate the OER activity of different catalysts loaded on the conductive nickel foam (NF), and the mass loading of 1 mg cm^−2^ is about 4 times that of RDE. The NiFeV nanofibers/NF electrode can achieve a very low overpotential of 294 mV at 500 mA cm^−2^ (Figure 5b). Figure 5c further summarized and compared the overpotentials of different working electrodes at a current density of 100 mA cm^−2^. To reach the current density of 100 mA cm^−2^, NiFeV nanofibers/KB and NiFeV nanofibers/NF only need the overpotentials of 305 and 252 mV, respectively. Moreover, CC is also employed as the conductive substrate due to its low price, large surface area, and good conductivity. When loaded on CC with the mass loading of 1 mg cm^−2^, the NiFeV nanofibers/CC electrode exhibits outstanding activity with low overpotentials of 181, 269, and 327 mV at the current density of 10, 100, and 500 mA cm^−2^ (Figure 5d and e). The NiFeV nanofibers/CC electrode demonstrates a small Tafel slope of 47 mV dec^−1^, which is much smaller than that of pure NiFeV and RuO_2_ (Figure 5f). Therefore, the conductivity and loading amount play a crucial role in OER catalysts (Figure S17, Supporting Information).

Furthermore, chronopotentiometry measurements with two current steps were conducted to evaluate the durability of the NiFeV nanofibers/CC. The NiFeV nanofibers/CC can function steadily at constant current densities of 20 and 100 mA cm^−2^. After running 80 000 s for each step, only a 7 mV shift was observed at 20 mA cm^−2^, and 72 mV for 100 mA cm^−2^ (Figure 5g). Compared with NiFeV nanofibers/CC, the bare CC presents almost no OER activity, indicating the negligible contribution of CC substrate to the oxygen evolution current (Figure S18, Supporting Information). Remarkably, compared to the currently reported catalysts, the NiFeV nanofibers/CC displays very excellent OER performances and ranks among the best (Figure 5h and Table S4, Supporting Information). The XPS characterization was conducted on the post reacted NiFeV nanofibers/CC sample to reveal the valence changes of metal elements during the OER. The result points to a slight increase in the binding energy for Ni, Fe, and V (Figure S19, Supporting Information), indicating an increase in the oxidation state, and activation process performed during the OER process.

**Figure 6:**
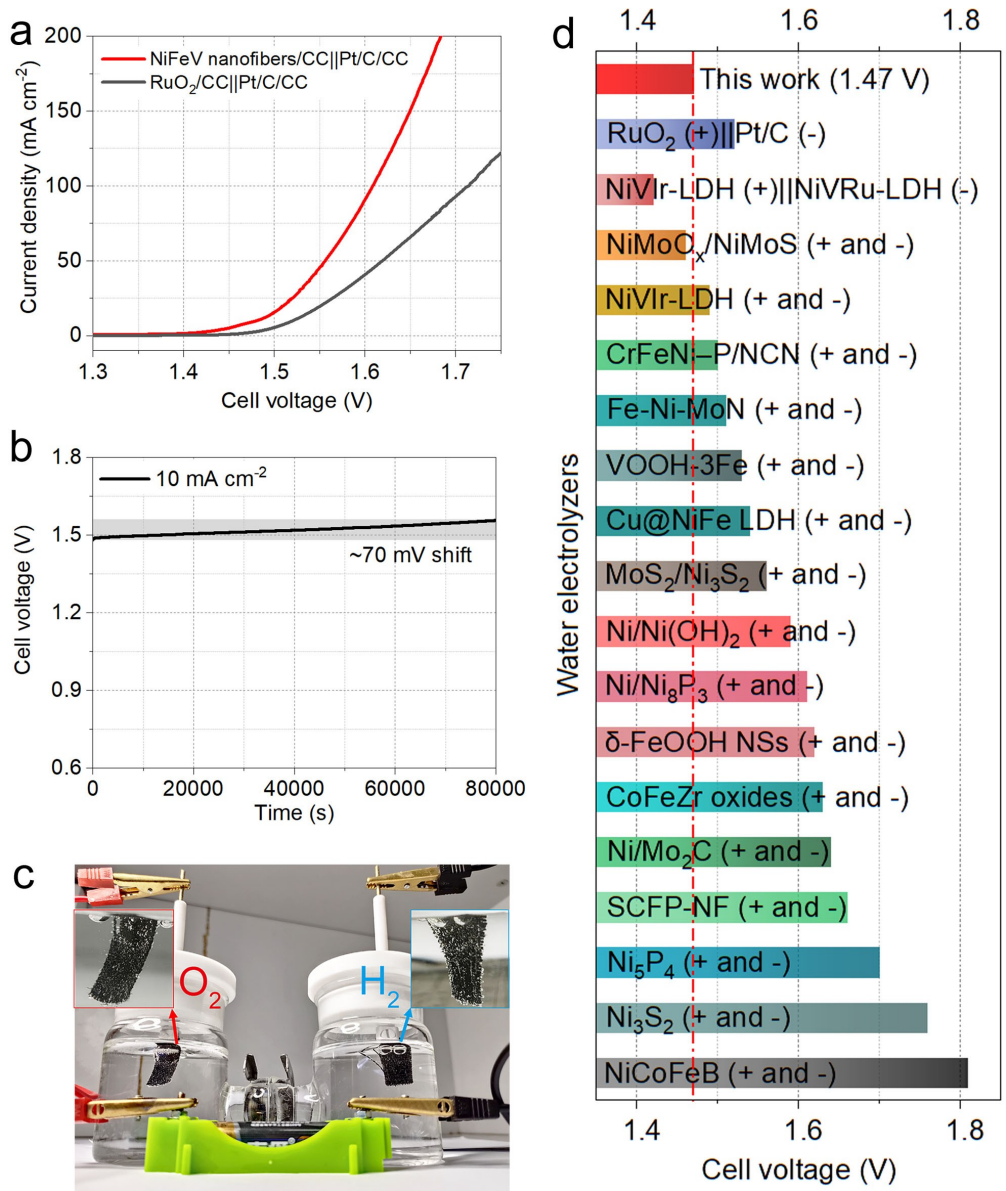
a) The polarization curves of NiFeV nanofibers/CC||Pt/C/CC and RuO_2_/CC||Pt/C/CC toward overall water splitting. b) Long‐time stability of NiFeV nanofibers/CC||Pt/C/CC for overall water splitting at 10 mA cm^−2^. c) The photo of electrolyzer driven by a 1.5 V AA battery. d) Comparison of the cell voltage at 10 mA cm^−2^ for the NiFeV nanofibers/CC||Pt/C/CC with other reported water electrolyzers.

To further evaluate the application potentials of this material, we also evaluated the overall water splitting performance in a two‐electrode system (1 M KOH) by using the NiFeV nanofibers/CC electrode as anode and commercial Pt/C supported on CC substrate (Pt/C/CC) as the cathode (the cell is named as NiFeV nanofibers/CC||Pt/C/CC). For comparison, the overall water splitting performance made from commercial catalysts RuO_2_/CC||Pt/C/CC has also been tested in 1 M KOH. The polarization curves are shown in Figure 6a, indicating that for driving a current density of 10 mA cm^−2^, the required voltage is as low as 1.47 V for the NiFeV nanofibers/CC||Pt/C/CC; while a voltage of 1.52 V is needed for the RuO_2_/CC||Pt/C/CC electrodes. Significantly, the NiFeV nanofibers/CC||Pt/C/CC based device shows excellent operation stability at 10 mA cm^−2^ for over 80 000 s without noticeable performance decline (Figure 6b). Benefiting from the excellent overall water splitting activity, the electrolyzer can also be driven by a 1.5 V AA battery at room temperature (Figure 6c and Supporting Information Movie 1). The recorded video clearly shows the formation of oxygen and hydrogen bubbles on the anode and cathode, respectively. Compared to most of currently reported water electrolyzers (Figure 6d and Table S5, Supporting Information), the cell voltage of NiFeV nanofibers/CC||Pt/C/CC at 10 mA cm^−2^ is much lower than those of NiVIr‐LDH||NiVIr‐LDH (1.49 V),^[29]^ VOOH‐3Fe||VOOH‐3Fe (1.53 V),^[30]^ Cu@NiFe LDH||Cu@NiFe LDH (1.54 V),^[31]^ MoS_2_/Ni_3_S_2_||MoS_2_/Ni_3_S_2_ (1.56 V),^[32]^ Ni/Ni(OH)_2_∥Ni/Ni(OH)_2_ (1.58 V),^[33]^ δ‐FeOOH NSs||δ‐FeOOH NSs (1.62 V),^[34]^ NiCoFeB|| NiCoFeB (1.81 V),^[35]^ etc.

Combining the above results, we can conclude that the interfacial atom‐substitution strategy achieves higher controllability on electronic structures of V‐doped NiFe electrocatalysts compared with the conventional one‐pot strategy. Although the OER active sites of NiFe catalysts are controversial, it is increasingly convinced that the high‐valence transition metals, especially Fe, can optimize the OER intermediates’ free energy, thus enhancing the catalytic activity and kinetics.[^10c^, ^13b^, ^14^, ^22a^] In our NiFeV nanofiber electrocatalyst, the abundant formed high‐valence Fe via a charge transfer from Fe to V played an important role in optimizing the OER performance, and the Fe−O−V−O−Ni bridge could be recognized as the active site.^[17a]^

## Conclusion

In summary, our findings on the synthesis of electrocatalytic oxygen‐evolving NiFeV nanofibers suggest that the interfacial atom‐substitution strategy offers an effective way to optimize the OER performance of NiFe catalysts and enhance the activity and stability for high‐performance OER catalysis. The electronic structure analysis demonstrated that the high oxidation state of Fe species could be stabilized in the NiFeV nanofibers via the partial electron transfer from Fe to V species. The electrocatalytic tests validated that NiFeV nanofiber exhibited much higher OER catalytic performance than commercial RuO_2_ and previously reported NiFeV compounds. We are convinced that applying such a substitutional growth strategy constitutes a new and promising approach to developing other efficient and durable non‐noble metal‐based OER catalysts.

## Conflict of interest

The authors declare no conflict of interest.

1

## Supporting information

As a service to our authors and readers, this journal provides supporting information supplied by the authors. Such materials are peer reviewed and may be re‐organized for online delivery, but are not copy‐edited or typeset. Technical support issues arising from supporting information (other than missing files) should be addressed to the authors.

Supporting Information

Supporting Information

## Data Availability

The data that support the findings of this study are available from the corresponding author upon reasonable request.
