# Supplementary material for: Interfacial Atom‐Substitution Engineered Transition‐Metal Hydroxide Nanofibers with High‐Valence Fe for Efficient Electrochemical Water Oxidation
Source: Angew Chem Int Ed Engl. 2022 Jan 28;61(13):e202115331. doi: 10.1002/anie.202115331 (PMC9306610; doi:10.1002/anie.202115331)
Supplement: Supplementary file 1 — Supporting Information [file ANIE-61-0-s002.pdf]

Angewandte Chemie
Eine Zeitschrift der Gesellschaft Deutscher Chemiker

Supporting Information

Interfacial Atom-Substitution Engineered Transition-Metal Hydroxide Nanofibers with High-Valence Fe for Efficient Electrochemical Water Oxidation

B. Zhang, Z. Wu, W. Shao, Y. Gao, W. Wang, T. Ma, L. Ma, S. Li, C. Cheng*, C. Zhao**

Author Contributions

B.Z., S.L., C.Z., and C.C. conceived the idea and designed the project. B.Z. performed the major experiments and analyzed the results. Z.W., W.S., Y.G., W.W., T.M., and L.M. assisted with the figure production, data analysis, and experiment design. B.Z., S.L., and C.C. wrote and edited the manuscript. S.L., C.C., and C.Z. supervised the whole project. All authors discussed the results and commented on the manuscript.

Experimental Section

1. Materials and Chemicals

Ethanol (C_2H_5OH, 99.7%, Aladdin), p-phthalic acid ($C_8H_6O_4$, 99%, Aladdin), nickel(II) nitrate hexahydrate ($Ni(NO_3)_2{\cdot}6H_2O$, 98%, Aladdin), ferrous sulfate heptahydrate ($FeSO_4{\cdot}7H_2O$, 99.0%, Aladdin), N, N-Dimethylformamide (DMF, C_3H_7NO, 99.5%, Aladdin), urea (CH_4N_2O, 99.5%, Aladdin), vanadium(IV) sulfate oxide hydrate ($VOSO_4{\cdot}xH_2O$, 99.9% metals basis, Alfa Aesar), iridium oxide powder (RuO_2, 99%, Alfa Aesar), Nafion (D520, 5 wt%, Alfa Aesar), and potassium hydroxide (KOH, 85%, Aladdin) were used as received. Ultrapure water was used for the preparation of all aqueous solutions.

2. Synthesis of V_3O_7 Nanofibers

2.0 mmol (0.43 g) of $VOSO_4{\cdot}xH_2O$ was dissolved in ultrapure water (10 mL) under ultrasonication. Then, 10 mL ultrapure water containing 2.0 mmol (0.32 g) of p-phthalic acid was poured into the above solution. After ultrasonic treatment for 30 min, the mixture was transferred into a 50 mL Teflon-lined stainless steel autoclave and reacted at 180 °C for 12 h. The green precipitate was collected and washed with ultrapure water and DMF solution several times and then dried under vacuum at 60 °C overnight.

3. Synthesis of NiFeV Nanofibers

20 mg of V_3O_7 nanofibers were dispersed in 6 mL of ethanol to form solution A. 50 mg of $FeSO_4{\cdot}7H_2O$, 150 mg of $Ni(NO_3)_2{\cdot}6H_2O$, and 200 mg of urea were dissolved into 4 mL of ultrapure water as solution B. Then, solution B was rapidly poured into solution A at room temperature as solution C. Finally, solution C was tightly sealed in a glass bottle and heated at 80 °C for 15 h without stirring. The product was washed with water and ethanol several times and then dried at 60 °C.

4. Characterization

Scanning electron microscopy (SEM) images were obtained by using a Thermo Fisher Scientific (FEI) Apreo S HiVoc. The gold coating was deposited with a layer of about 1 nm. The

transmission electron microscope (TEM) was performed by Tecnai G2 F20 S-TWIN operated at 200 kV. X-ray diffraction (XRD) measurements were characterized by Rigaku Ultima IV with Cu Kα irradiation. X-ray photoelectron spectra (XPS) measurements were performed on K-Alpha™+ X-ray Photoelectron Spectrometer System (Thermo Scientific) with Hemispheric 180° dual-focus analyzer with 128-channel detector and a monochromatic Al Kα irradiation. X-ray absorption spectra (XAS) were collected on the beamline BL07A1 in NSRRC (National Center for Synchrotron Radiation Research). The radiation by scanning a Si (111) double-crystal monochromator.

5. Electrochemical Measurements:

In a typical preparation of catalyst ink, 10 mg of each catalyst was blended with 1.0 mL Nafion ethanol solution (0.5 wt%) in an ultrasonic bath for 30 min. The carbon cloth (CC) and Ni foam (NF) were ultrasonically cleaned by acetone, ethyl alcohol, and ultrapure water in sequence for 20 min. Subsequently, the CC was submerged in a 2 M H_2SO_4 solution for another 12 h, and the nickel foam was sonicated in 2 M HCl for 20 min. To remove any additional acid, the CC and NF were rinsed with ultrapure water several times and then left dry in the air. Then a fixed volume of catalyst ink (10 mg mL^{-1}) was pipetted onto the NF (loading: 1 mg cm^{-2}), CC (loading: 1 mg cm^{-2}), and glassy carbon electrode (loading: 0.255 mg cm^{-2}).

All the electrochemical measurements were carried out in a conventional three-electrode cell using the Gamry reference 600 workstation (Gamry, USA) at room temperature. A commercial reversible hydrogen electrode (RHE) was used as the reference electrode, and the graphite rod was used as the counter electrode. The Ag/AgCl reference electrode calibrated with RHE in 1 M KOH was used as a reference electrode for long-time stability measurement. NF (1.0 × 1.0 cm^2) and CC (1.0 × 1.0 cm^2), as well as glassy carbon electrode with an area of 0.196 cm^2, served as the substrate for the working electrode to evaluate the OER activities of various catalysts. The electrochemical experiments were conducted in an Ar saturated 1 M KOH electrolyte. The rotating disk electrode (RDE) measurements were conducted at a rotating speed of 1600 rpm with a sweep rate of 10 mV s^{-1}. The OER polarization curves of different catalysts with real-time iR corrected by Gamry reference 600 potentiostats at a resistance of 4.4 ohms.

The electrochemically active surface area was estimated by measuring the capacitance of the double layer at the solid-liquid interface with cyclic voltammetry. The measurement was performed

in a potential window of 0.82–0.92 V versus RHE, where the Faradic current on the working electrode was negligible. The series of scan rates ranging from 50 to 300 mV s^{-1} was applied to build a plot of the charging current density differences against the scan rate at a fixed potential of 0.87 V. The slope of the obtained linear curve was twice of the double-layer capacitance (C_{dl}).[1]

Electrochemical impedance spectroscopy (EIS) was carried out with a potentiostatic EIS method with a DC voltage of 1.495 V versus RHE in an Ar saturated 1.0 M KOH electrolyte from 100 kHz to 0.1 Hz with a 10 mV AC potential at 1600 rpm. The stability tests for the catalysts were conducted using chronopotentiometry at the constant working current densities of 10, 20, and 100 mA cm^{-2}.

The TOF values were calculated as the number of oxygen molecules evolved per active site per second based on the following equation:

$$TOF = \frac{J * A}{4F * m}$$

Where J is the current density (A cm^{-2}) at the overpotential of 350 mV, A is the effective surface geometric area of the working electrode (0.196 cm^{-2}), F is the Faraday constant, and n is the number of the active metal on the electrode. The TOFs data was calculated based on the weight content (from XPS) of the Fe in the catalysts.

Supplementary Images:

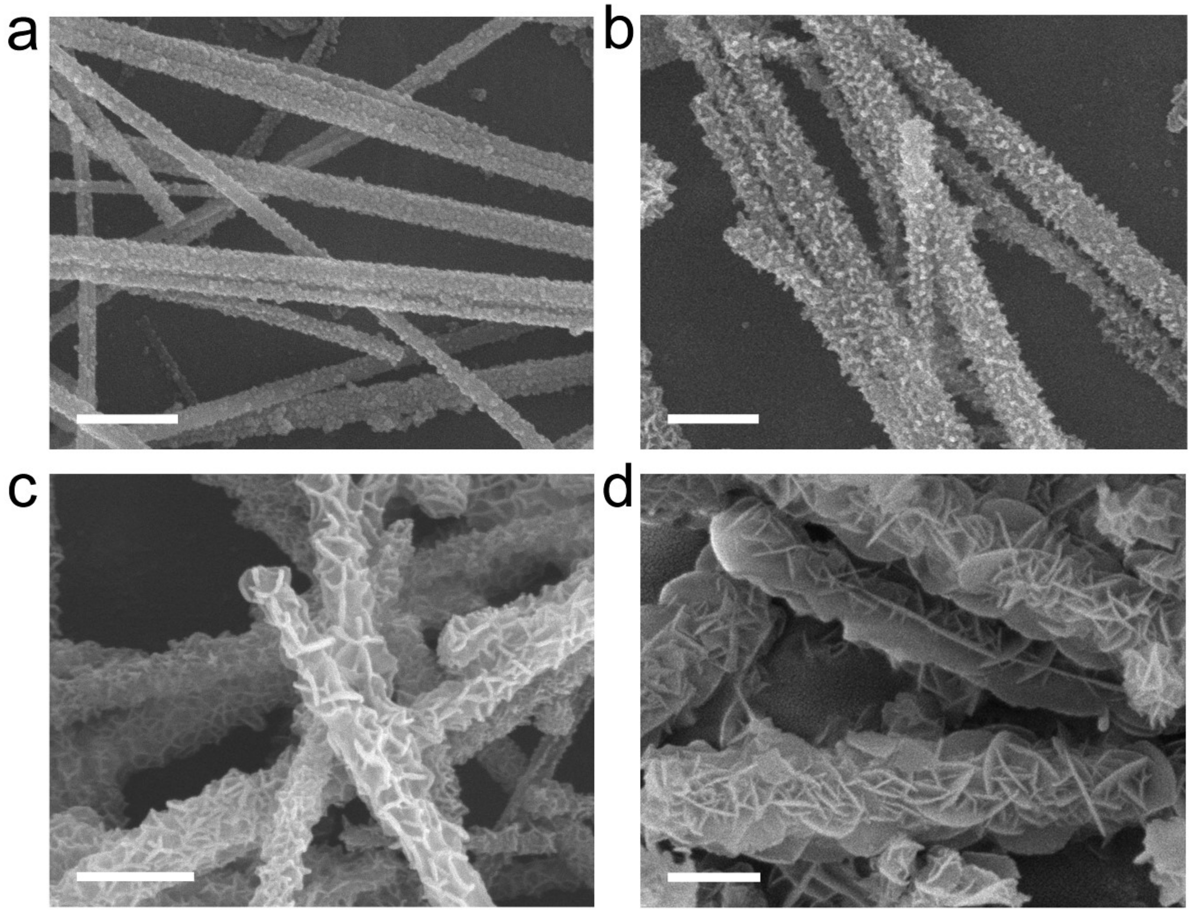

Figure S1. SEM images of NiFeV hydroxides supported on V_3O_7 prepared with different reaction times: (a) 1, (b) 5, (c) 10, (d) 24 h. Scale bars: a-d 500 nm.

In Figure S1a, only a small amount of nanosheets was loaded on the outside of V_3O_7 since the beginning due to the incomplete deposition. With the further extension of synthesis time to 5-10 h, uniform and thin hydroxide nanosheets can be observed on the V_3O_7 nanofibers (Figure S1b and S1c). However, when the reaction time exceeds 24 h, excessive growth of hydroxide nanosheets can be observed with large size and thickness (Figure S1d). The SEM images indicate that reaction time significantly influences the density of NiFeV nanosheets on V_3O_7.

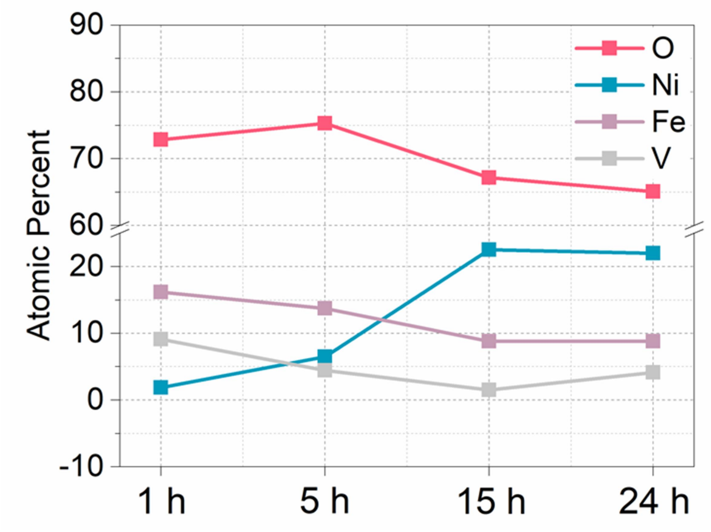

Figure S2. Atomic ratio variation of NiFeV supported on V_3O_7 prepared with different times.

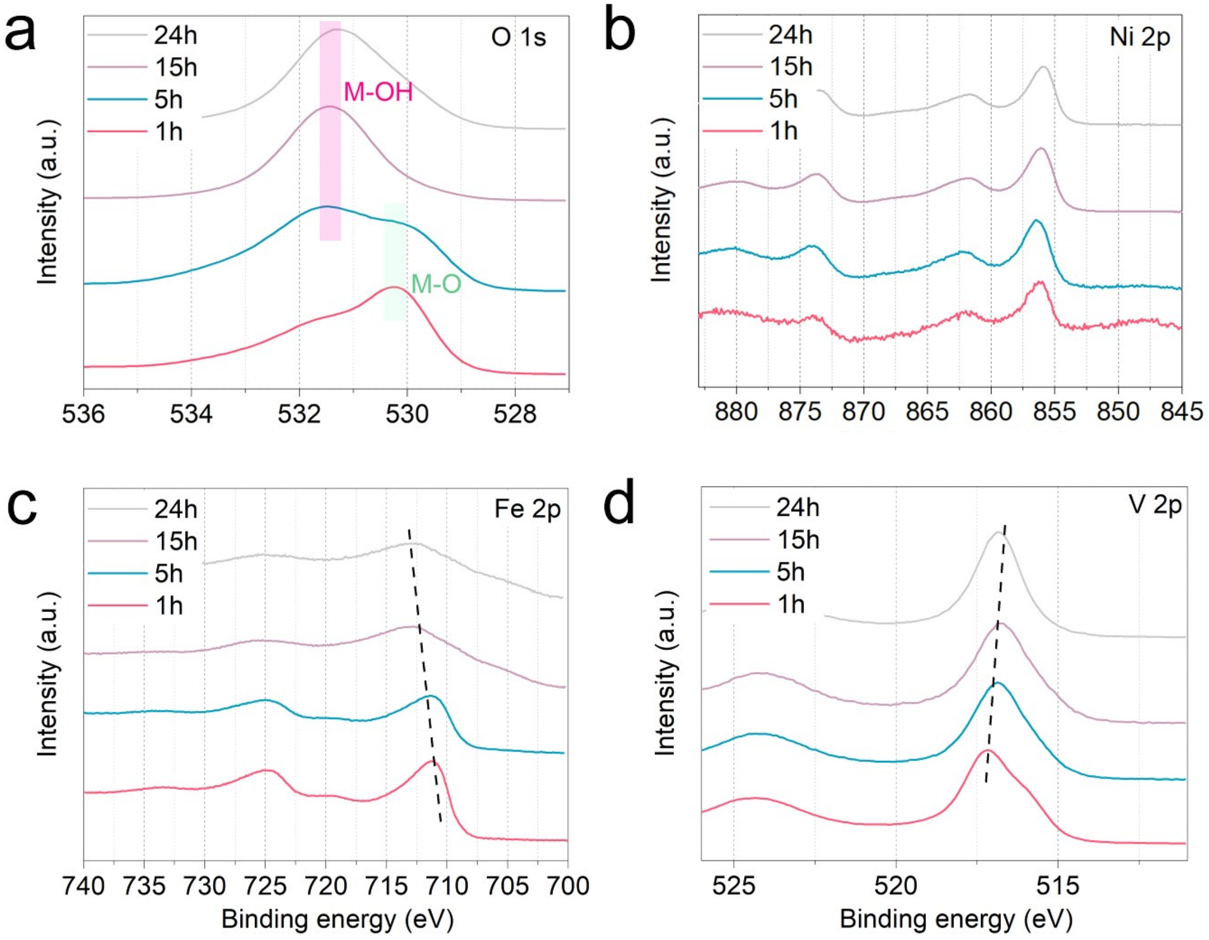

Figure S3. XPS spectra of NiFeV nanofibers prepared with different times. (a) O 1*s*, (b) Ni 2*p*, (c) Fe 2*p*, and (d) V 2*p*.

To investigate the evolution of electronic structures during the preparation process, X-ray photoelectron spectroscopy (XPS) measurements at different reaction stages were conducted. Since solid V_3O_7 is the only source of V ion release, the NiFe/V ratio on the sample surface increases with the prolonging of reaction times (< 15h) (**Figure S2**). As the synthesis time was extended to 24 h, the contents of V substitution will further increase. As seen in **Figure S3a**, the peaks at 530.4 and 531.4 eV originate from oxide species and oxygen ions in NiFeV nanofibers, respectively. The signal of M-O peak is retained after the 5 h reaction, indicating an incomplete surface etching of the V_3O_7. After another 10 h of reaction, M-O signal can no longer be detected on the skeleton surface, manifesting a complete integrating of the highly active NiFeV hydroxide nanosheets on V_3O_7. The high-resolution Ni 2*p*, Fe 2*p*, and V 2*p* XPS scans of different samples were further investigated to identify the electronic structures of Ni, Fe, and V elements (**Figure S3b-d**). With the reaction time extended to 15 h, the iron atom was gradually oxidized to positive trivalence. It is worthy of note that the V 2*p* peaks are shifted to lower binding energy with increased time, indicating that the V species on the surface of V_3O_7 were gradually etched and doped into hydroxides. These XPS results further revealed that with the prolonging of reaction time, the released V species steadily coprecipitate with Ni and Fe in solution to form NiFeV hydroxide gradually. Therefore, the reaction time plays an imperative role in controlling the contents of V substitution during the hydrolysis reactions.

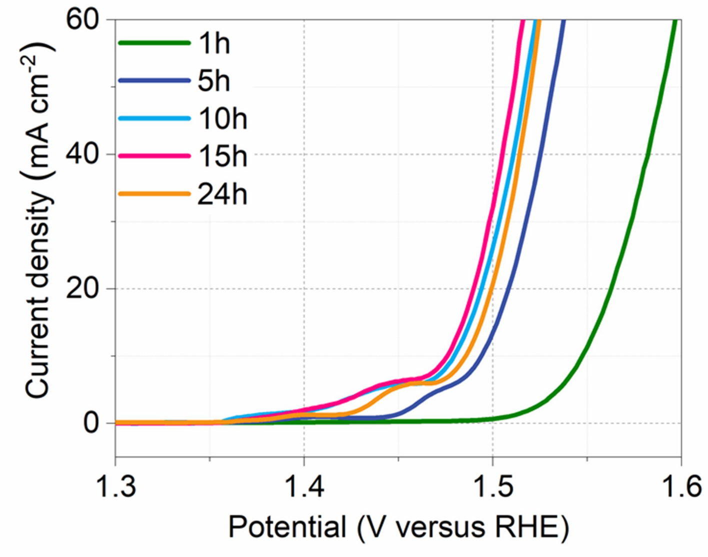

Figure S4. LSV curves of NiFeV nanofibers prepared with different times.

The optimal reaction time is determined to be 15 h by electrochemical OER tests (**Figure S4**); the overpotential increases first and then declines with the prolonging of treatment time, where the maximum value occurs at 15 h.

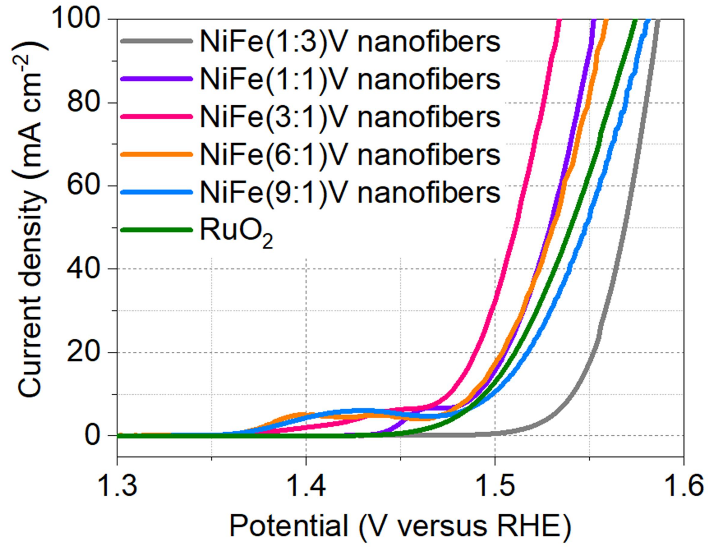

Figure S5. LSV curves of NiFeV nanofibers prepared with different Fe/Ni feed weight ratios.

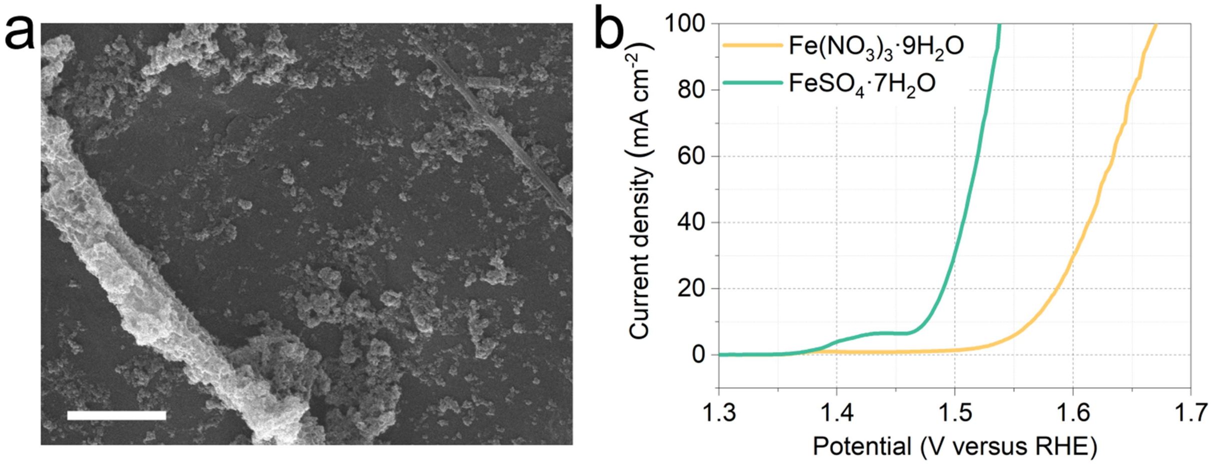

Figure S6. (a) SEM image of NiFeV nanofibers with iron (III) nitrate nonahydrate as Fe source. Scale bars: 1 μm. (b) LSV curves of samples with different iron salts as precursors.

The pH values of the dissolved iron (III) nitrate nonahydrate and iron (II) sulfate heptahydrate are 1.84 (5 mg/mL) and 4.45 (5 mg/mL), respectively. When iron (III) nitrate nonahydrate replaces iron (II) sulfate heptahydrate as the iron source, the fiber structure of the V_3O_7 is destroyed, and the catalytic performance of OER decreases. This is due to the stronger Lewis acid condition for iron (III) nitrate nonahydrate.

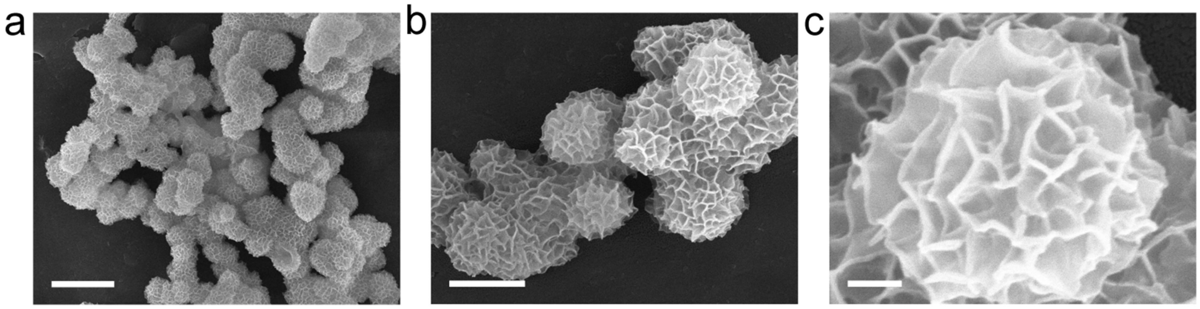

Figure S7. SEM images of NiFeV hydroxides at different magnifications. Scale bars: a 2 μm; b 1 μm; c 200 nm.

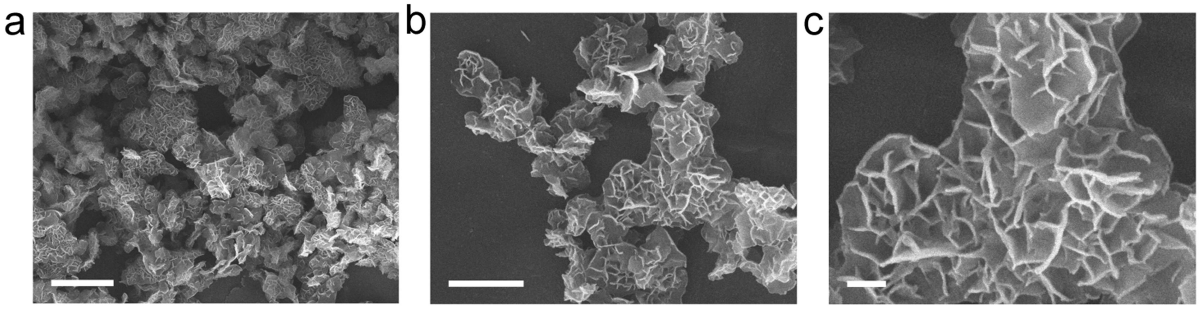

Figure S8. SEM images of NiFe hydroxides at different magnifications. Scale bars: a 2 μm; b 1 μm; c 200 nm.

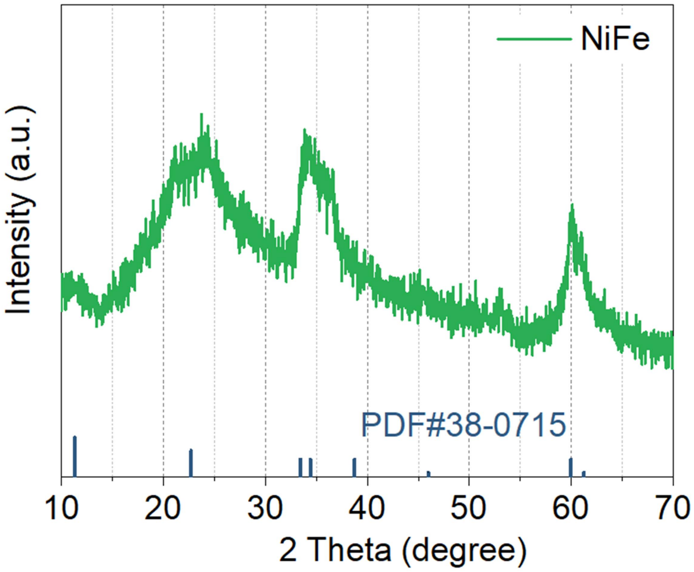

Figure S9. XRD pattern of NiFe hydroxides.

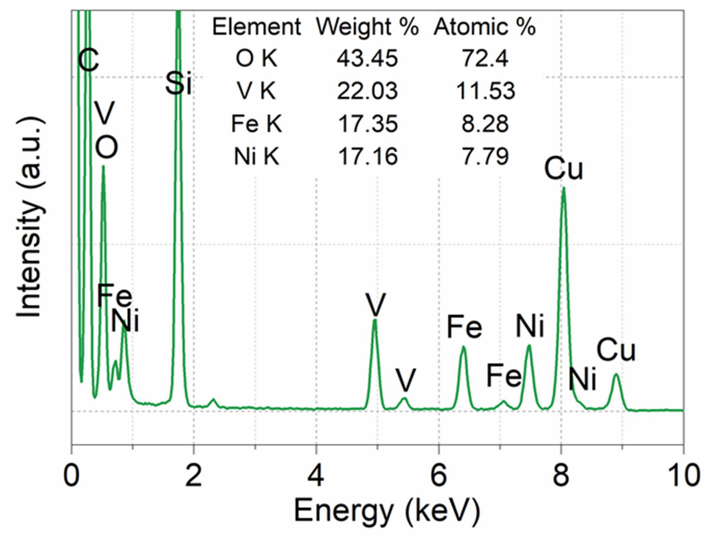

Figure S10. EDS pattern of NiFeV nanofibers, where the C, Cu, and Si signals come from the sample holder and contamination.

Table S1. The surface atomic composition determined by XPS.

Samples	O	Fe	Ni	V
NiFeV nanofibers	67.13	8.79	22.58	1.5
NiFeV	67.35	11.5	19.53	1.62
NiFe	67.78	10.85	21.37	

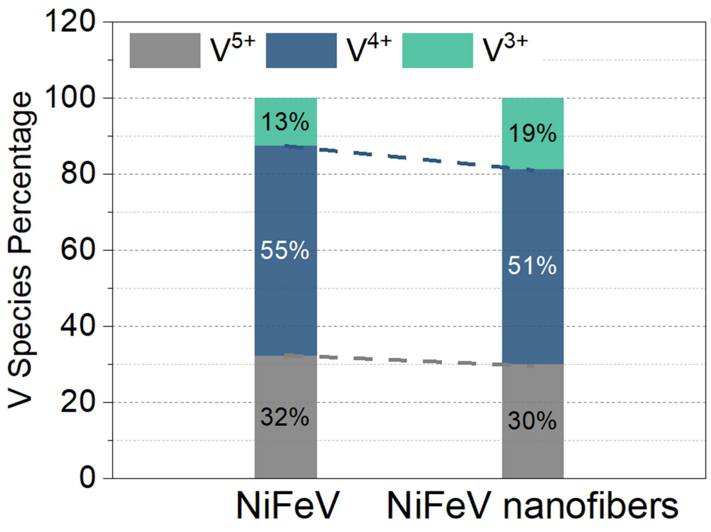

Figure S11. Valence distribution of V for different samples.

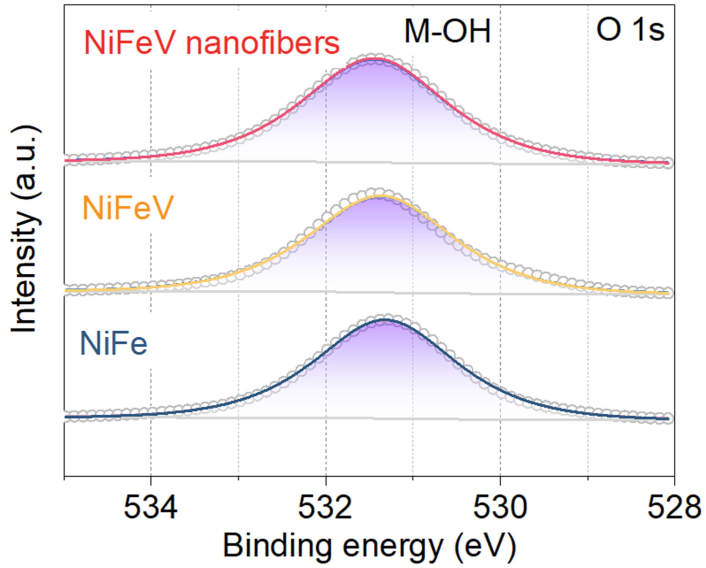

Figure S12. O 1s XPS spectra of NiFeV nanofibers, NiFeV, and NiFe.

Table S2. Comparison of OER catalytic parameters of NiFeV nanofibers with previously reported catalysts.

Catalyst	Electrolyte	Overpotential (mV) at 10 mA cm^{-2}	Tafel slope (mV dec^{-1})	Mass loading (mg cm^{-2})	Reference
NiFeV nanofibers	1 M KOH	263	47	0.255	This work
$Fe_5Co_4Ni_{20}Se_{36}B_x$	1 M KOH	280	60	0.255	[2]
CoFe LDHs-Ar	1 M KOH	266	38		[3]
Ni-Fe LDH hollow prisms	1 M KOH	280	49	0.160	[4]
Bulk CoOOH	1 M KOH	370	69	0.255	[5]
$Sr_3FeCoO_{7-\delta}$	1 M KOH	343	63	0.283	[6]
Holey $Ni(OH)_2$	1 M KOH	335	65		[7]
FeNiP/NCH	1 M KOH	340	68	0.500	[8]
Co-Fe-P-Se/NC	1 M KOH	270	42	0.350	[9]
$Co(OH)_2/Co_3O_4$	1 M KOH	281	52.7	0.283	[10]
$SnCo_{0.9}Fe_{0.1}$-$(OH)_6$	1 M KOH	300	42.3	0.485	[11]
LDH NiFe	1 M KOH	300	38	0.07	[12]

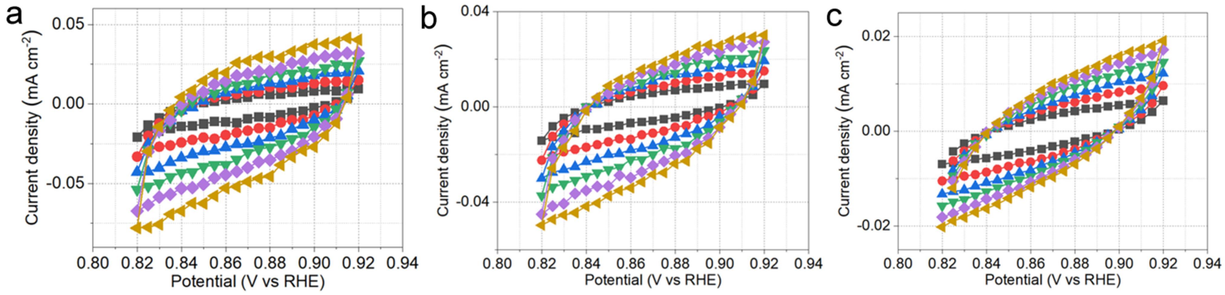

Figure S13. Cyclic voltammograms of (a) NiFeV nanofibers, (b) NiFeV, and (c) NiFe in 1 M KOH at different scan rates (50, 100, 150, 200, 250, and 300 mV s^{-1}) in the non-Faradaic potential region (0.82–0.92 V versus RHE).

Table S3. Comparison of the C_{dl}, ECSA, mass activity, and specific activity for as-prepared catalysts.

Samples	C_{dl} ($mF\cdot cm^{-2}$)	ECSA	Mass activity ($A\cdot g^{-1}$)	Specific activity ($mA\cdot cm^{-2}$)
NiFeV nanofibers	0.129	2.15	93.6	11.11
NiFeV	0.069	1.15	13.36	2.96
NiFe	0.027	0.45	14.31	8.11

The C_{dl} can be calculated from the scan rates with capacitive current densities obtained from CV curves (Figure S13). ECSA was calculated by C_{dl} dividing the capacitance of these electrocatalysts with a specific capacitance (0.06 mF cm^{-2}).[13] The mass activity was calculated via currents normalized by the loading mass of electrocatalysts. Specific activity was calculated via current densities normalized by the ECSAs of electrocatalysts.

The mass and specific activities were obtained from the current densities at 1.53 V vs. RHE.

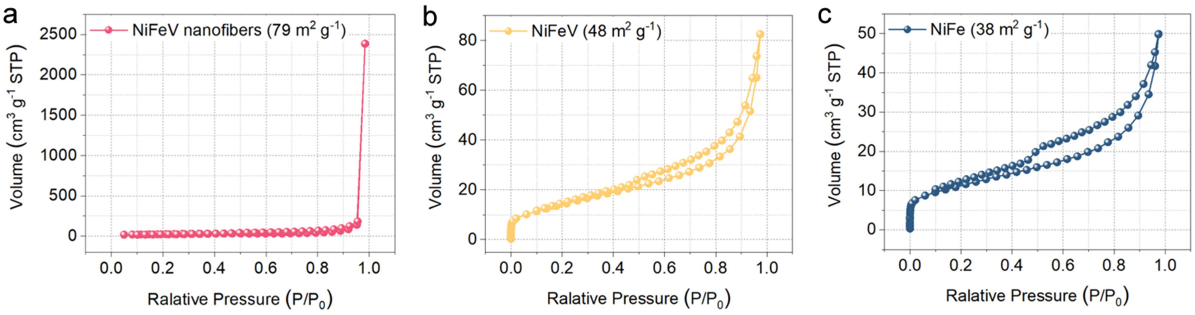

Figure S14. N_2 adsorption/desorption isotherms of NiFeV nanofibers, NiFeV, and NiFe.

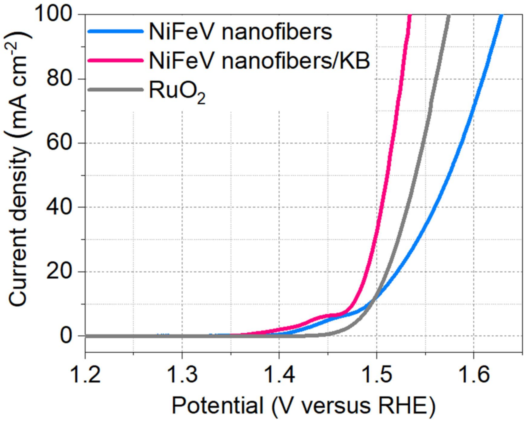

Figure S15. LSV curves of NiFeV nanofibers, NiFeV nanofibers/KB, and RuO_2.

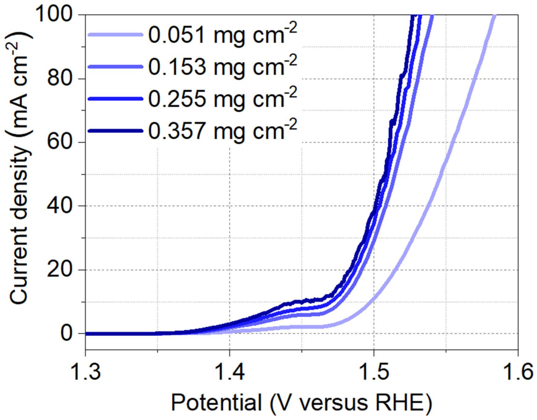

Figure S16. LSV curves of NiFeV nanofibers/KB with different loading amounts.

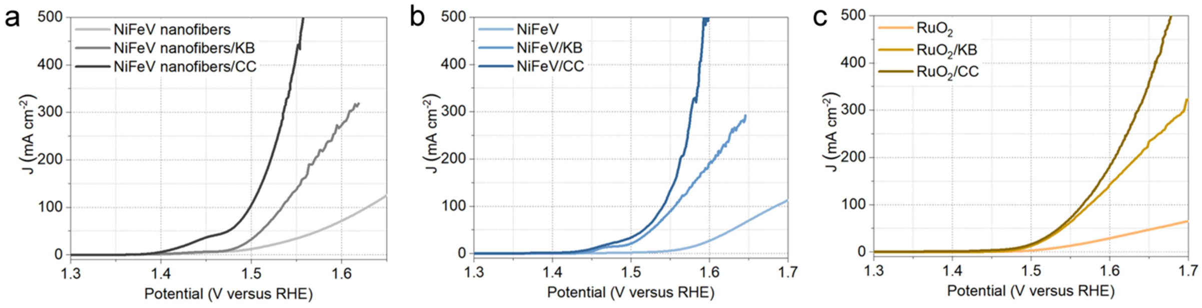

Figure S17. Comparison of polarization curves for (a) NiFeV nanofibers, (b) NiFeV, and (c) NiFe.

Table S4. The electrocatalytic performances of NiFeV nanofibers/CC compared with some state-of-the-art catalytic electrodes. (CFP: carbon fiber paper; CC: carbon cloth; CP: carbon paper; CF: carbon foam)

Catalyst	Substrate	Electrolyte	Overpotential (mV) at 10 mA cm^{-2}	Tafel slope (mV dec^{-1})	Mass loading (mg cm^{-2})
NiFeV nanofibers (This work)	CC	1 M KOH	181	47	1.0
M-PCBN[2]	CC	1 M KOH	232	32	0.93
Co_3O_4 nanosheet[3]	CP	1 M KOH	270	46	0.1
Co-Ni_3N[4]	CC	1 M KOH	307	57	2.91
NiCoP/CC[5]	CC	1 M KOH	242	64.2	2
Ir-NiO[6]	CC	1 M KOH	215	38	1.2
NiFexSn-A[7]	CC	1 M KOH	260	50	
α-$Ni(OH)_2$/CeO_2[8]	CF	1 M KOH	240	57	
Fe-Mn-O NSs/CC[9]	CC	1 M KOH	273	63.9	1.6
Ru/CoFe-LDHs[10]	CFP	1 M KOH	198	39	1.0
$Ni_3Fe_{0.5}V_{0.5}$[11]	CFP	1 M KOH	200	39	0.2
Ag@$Co(OH)_x$[12]	CC	1 M KOH	250	76	1.5
$Co_{1-x}Fe_xSe_2$-0.6[13]	Ni foam	1 M KOH	217	41	2.1
$Ni_xFe_{1-x}Se_2$-DO[14]	Ni foam	1 M KOH	195	28	
Ni-Fe NP[15]	Ni foam	1 M KOH	210	53	2.5
NiFe LDH/Ni_2P[16]	Ni foam	1 M KOH	205	32	1.0
γ-FeOOH/NF-6M[17]	Ni foam	1 M KOH	286	51	0.5
CoNiP@NiFe LDH[18]	Ni foam	1 M KOH	216	45	
NiFeRu LDH[19]	Ni foam	1 M KOH	225	32.4	1.2
N-CNTs/$Ni(OH)_2$[20]	Ni foam	1 M KOH	254	84	0.8
G-FeCoW[21]	Au foam	1 M KOH	191		0.21
Co/Ni$(BDC)_2$TED[22]	Cu foam	1 M KOH	260	76	3.0

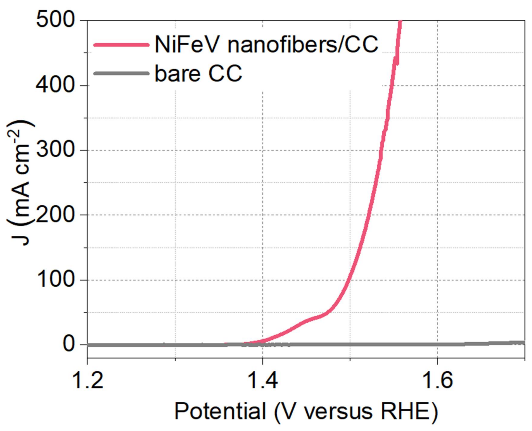

Figure S18. LSV curves of NiFeV nanofiber/CC and bare CC.

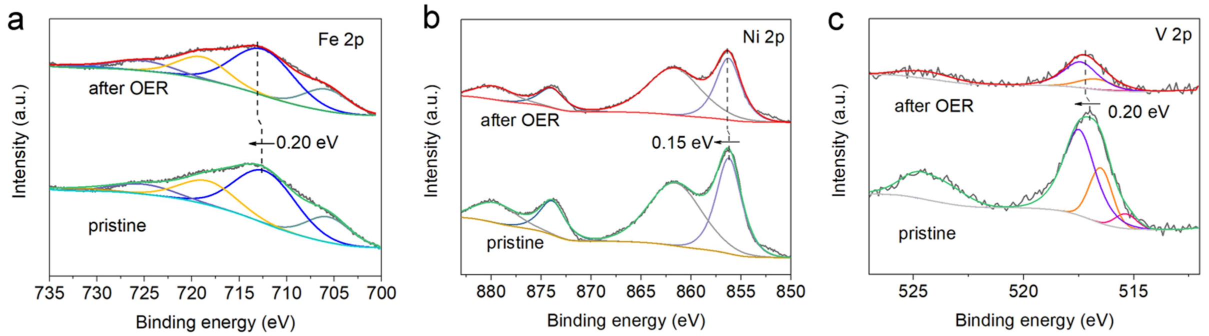

Figure S19. XPS spectra of (a) Fe 2*p*, (b) Ni 2*p*, and (c) V 2*p* for the NiFeV nanofibers/CC sample after OER tests in 1 M KOH.

Supplementary Table 5. Comparison of water splitting activity of NiFeV/V_3O_7/CC||Pt/C/CC cell in this work with other reported electrocatalysts in 1 M alkaline solution. (CFC: carbon fiber cloth, CP: carbon paper)

Catalyst	Substrate	Cell voltage (V) at 10 mA cm^{-2}	Electrolyte	Reference
NiFeV nanofibers||Pt/C/CC (This work)	CC	1.47	1 M KOH	
MoS_2/Ni_3S_2||MoS_2/Ni_3S_2	Ni foam	1.56	1 M KOH	[34]
Cu@NiFe LDH||Cu@NiFe LDH	Cu foam	1.54	1 M KOH	[35]
δ-FeOOH NSs||δ-FeOOH NSs	Ni foam	1.62	1 M KOH	[36]
IFONFs-45||IFONFs-45	Fe foil	1.58	1 M KOH	[37]
$Fe_{0.09}Co_{0.13}$-$NiSe_2$||$Fe_{0.09}Co_{0.13}$-$NiSe_2$	CFC	1.52	1 M KOH	[38]
NiVIr-LDH||NiVIr-LDH	Ni foam	1.49	1 M KOH	[39]
VOOH-3Fe||VOOH-3Fe	Ni foam	1.53	1 M KOH	[40]
$Ni/Ni(OH)_2$‖$Ni/Ni(OH)_2$	CFP	1.59	1 M KOH	[41]
Ni_3S_2@MoS_2/FeOOH||Ni_3S_2@MoS_2/FeOOH	Ni foam	1.57	1 M KOH	[42]
NiCoFeB||NiCoFeB	CFP	1.81	1 M KOH	[43]
NiVIr-LDH||NiVRu-LDH	Ni foam	1.42	1 M KOH	[44]

NiMoOx/NiMoS||NiMoOx/NiMoS	Ni foam	1.46	1 M KOH	[45]
CrFeNi–P/NCN||CrFeNi–P/NCN	Ni foam	1.50	1 M KOH	[46]
Fe-Ni-MoN||Fe-Ni-MoN	CP	1.51	1 M KOH	[47]
Ni/Ni_8P_3||Ni/Ni_8P_3	Ni foam	1.61	1 M KOH	[48]
CoFeZr oxides||CoFeZr oxides	Ni foam	1.63	1 M KOH	[49]
Ni/Mo_2C||Ni/Mo_2C	Ni foam	1.64	1 M KOH	[50]
SCFP-NF||SCFP-NF	Ni foam	1.66	1 M KOH	[51]
Ni_5P_4||Ni_5P_4	Ni foam	1.70	1 M KOH	[52]
Ni_3S_2||Ni_3S_2	Ni foam	1.76	1 M KOH	[53]

References

[1] S. Hao, M. Liu, J. Pan, X. Liu, X. Tan, N. Xu, Y. He, L. Lei, X. Zhang, *Nat. Commun.* **2020**, *11*, 5368.

[2] Y. Zuo, D. Rao, S. Ma, T. Li, Y. H. Tsang, S. Kment, Y. Chai, *ACS Nano* **2019**, *13*, 11469-11476.

[3] Y. Wang, Y. Zhang, Z. Liu, C. Xie, S. Feng, D. Liu, M. Shao, S. Wang, *Angew. Chem. Int. Ed.* **2017**, *56*, 5867-5871.

[4] L. Yu, J. F. Yang, B. Y. Guan, Y. Lu, X. W. Lou, *Angew. Chem. Int. Ed.* **2018**, *57*, 172-176.

[5] S. Song, H. Bao, X. Lin, X.-L. Du, J. Zhou, L. Zhang, N. Chen, J. Hu, J.-Q. Wang, *J. Energy Chem.* **2020**, *42*, 5-10.

[6] K. Xu, F. Song, J. Gu, X. Xu, Z. Liu, X. Hu, *J. Mater. Chem. A* **2018**, *6*, 14240-14245.

[7] X. Kong, C. Zhang, S. Y. Hwang, Q. Chen, Z. Peng, *Small* **2017**, *13*, 1700334.

[8] Y.-S. Wei, M. Zhang, M. Kitta, Z. Liu, S. Horike, Q. Xu, *J. Am. Chem. Soc.* **2019**, *141*, 7906-7916.

[9] H. Wu, J. Wang, J. Yan, Z. Wu, W. Jin, *Nanoscale* **2019**, *11*, 20144-20150.

[10] H. Xu, J. Wei, M. Zhang, C. Liu, Y. Shiraishi, C. Wang, Y. Du, *Nanoscale* **2018**, *10*, 18468-18472.

[11] D. Chen, M. Qiao, Y. R. Lu, L. Hao, D. Liu, C. L. Dong, Y. Li, S. Wang, *Angew. Chem. Int. Ed.* **2018**, *57*, 8691-8696.

[12] F. Song, X. Hu, *Nat. Commun.* **2014**, *5*, 1-9.

[13] J. Jiang, F. Sun, S. Zhou, W. Hu, H. Zhang, J. Dong, Z. Jiang, J. Zhao, J. Li, W. Yan, M. Wang, *Nat. Commun.* **2018**, *9*, 2885.

[14] W. Zhang, Y. Wang, H. Zheng, R. Li, Y. Tang, B. Li, C. Zhu, L. You, M.-R. Gao, Z. Liu, S.-H. Yu, K. Zhou, *ACS Nano* **2020**, *14*, 1971-1981.

[15] L. Dai, Q. Qin, X. Zhao, C. Xu, C. Hu, S. Mo, Y. O. Wang, S. Lin, Z. Tang, N. Zheng, *ACS Cent. Sci.* **2016**, *2*, 538-544.

[16] C. Zhu, A.-L. Wang, W. Xiao, D. Chao, X. Zhang, N. H. Tiep, S. Chen, J. Kang, X. Wang, J. Ding, J. Wang, H. Zhang, H. J. Fan, *Adv. Mater.* **2018**, *30*, 1705516.

[17] C. Du, L. Yang, F. Yang, G. Cheng, W. Luo, *ACS Catal.* **2017**, *7*, 4131-4137.

[18] Q. Wang, X. Huang, Z. L. Zhao, M. Wang, B. Xiang, J. Li, Z. Feng, H. Xu, M. Gu, *J. Am. Chem. Soc.* **2020**, *142*, 7425-7433.

[19] M. Chen, S. Lu, X. Z. Fu, J. L. Luo, *Adv. Sci.* **2020**, *7*, 1903777.

[20] Z. Liu, N. Li, H. Zhao, Y. Zhang, Y. Huang, Z. Yin, Y. Du, *Chem. Sci.* **2017**, *8*, 3211-3217.

[21] Y. Teng, X.-D. Wang, J.-F. Liao, W.-G. Li, H.-Y. Chen, Y.-J. Dong, D.-B. Kuang, *Adv. Funct. Mater.* **2018**, *28*, 1802463.

[22] P. Li, M. Wang, X. Duan, L. Zheng, X. Cheng, Y. Zhang, Y. Kuang, Y. Li, Q. Ma, Z. Feng, W. Liu, X. Sun, *Nat. Commun.* **2019**, *10*, 1711.

[23] Z. Zhang, X. Li, C. Zhong, N. Zhao, Y. Deng, X. Han, W. Hu, *Angew. Chem. Int. Ed.* **2020**, *59*, 7245-7250.

[24] J.-Y. Zhang, L. Lv, Y. Tian, Z. Li, X. Ao, Y. Lan, J. Jiang, C. Wang, *ACS Appl. Mater. Interfaces* **2017**, *9*, 33833-33840.

[25] X. Xu, F. Song, X. Hu, *Nat. Commun.* **2016**, *7*, 1-7.

[26] B. H. R. Suryanto, Y. Wang, R. K. Hocking, W. Adamson, C. Zhao, *Nat. Commun.* **2019**, *10*, 5599.

[27] F.-S. Zhang, J.-W. Wang, J. Luo, R.-R. Liu, Z.-M. Zhang, C.-T. He, T.-B. Lu, *Chem. Sci.* **2018**, *9*, 1375-1384.

[28] K. Wang, H. Du, S. He, L. Liu, K. Yang, J. Sun, Y. Liu, Z. Du, L. Xie, W. Ai, W. Huang, *Adv. Mater.* **2021**, *33*, 2005587.

[29] L. Zhou, S. Jiang, Y. Liu, M. Shao, M. Wei, X. Duan, *ACS Appl. Energy Mater.* **2018**, *1*, 623-631.

[30] G. Chen, T. Wang, J. Zhang, P. Liu, H. Sun, X. Zhuang, M. Chen, X. Feng, *Adv. Mater.* **2018**, *30*, 1706279.

[31] J. Wu, J. Subramaniam, Y. Liu, D. Geng, X. Meng, *J. Alloys Compd.* **2018**, *731*, 766-773.

[32] B. Zhang, X. Zheng, O. Voznyy, R. Comin, M. Bajdich, M. García-Melchor, L. Han, J. Xu, M. Liu, L. Zheng, F. P. García de Arquer, C. T. Dinh, F. Fan, M. Yuan, E. Yassitepe, N. Chen, T. Regier, P. Liu, Y. Li, P. De Luna, A. Janmohamed, H. L. Xin, H. Yang, A. Vojvodic, E. H. Sargent, *Science* **2016**, *352*, 333-337.

[33] D.-J. Li, Q.-H. Li, Z.-G. Gu, J. Zhang, *J. Mater. Chem. A* **2019**, *7*, 18519-18528.

[34] J. Zhang, T. Wang, D. Pohl, B. Rellinghaus, R. Dong, S. Liu, X. Zhuang, X. Feng, *Angew. Chem. Int. Ed.* **2016**, *55*, 6702-6707.

[35] L. Yu, H. Zhou, J. Sun, F. Qin, F. Yu, J. Bao, Y. Yu, S. Chen, Z. Ren, *Energy Environ. Sci.* **2017**, *10*, 1820-1827.

[36] B. Liu, Y. Wang, H.-Q. Peng, R. Yang, Z. Jiang, X. Zhou, C.-S. Lee, H. Zhao, W. Zhang, *Adv. Mater.* **2018**, *30*, 1803144.

[37] X. Fan, Y. Liu, S. Chen, J. Shi, J. Wang, A. Fan, W. Zan, S. Li, W. A. Goddard, X.-M. Zhang, *Nat. Commun.* **2018**, *9*, 1809.

[38] Y. Sun, K. Xu, Z. Wei, H. Li, T. Zhang, X. Li, W. Cai, J. Ma, H. J. Fan, Y. Li, *Adv. Mater.* **2018**, *30*, 1802121.

[39] S. Li, C. Xi, Y.-Z. Jin, D. Wu, J.-Q. Wang, T. Liu, H.-B. Wang, C.-K. Dong, H. Liu, S. A. Kulinich, X.-W. Du, *ACS Energy Lett.* **2019**, *4*, 1823-1829.

[40] J. Zhang, R. Cui, C. Gao, L. Bian, Y. Pu, X. Zhu, X. a. Li, W. Huang, *Small* **2019**, *15*, 1904688.

[41] L. Dai, Z. N. Chen, L. Li, P. Yin, Z. Liu, H. Zhang, *Adv. Mater.* **2020**, *32*, 1906915.

[42] M. Zheng, K. Guo, W.-J. Jiang, T. Tang, X. Wang, P. Zhou, J. Du, Y. Zhao, C. Xu, J.-S. Hu, *Appl. Catal., B* **2019**, *244*, 1004-1012.

[43] Y. Li, B. Huang, Y. Sun, M. Luo, Y. Yang, Y. Qin, L. Wang, C. Li, F. Lv, W. Zhang, S. Guo, *Small* **2019**, *15*, 1804212.

[44] D. Wang, Q. Li, C. Han, Q. Lu, Z. Xing, X. Yang, *Nat. Commun.* **2019**, *10*, 2899.

[45] P. Zhai, Y. Zhang, Y. Wu, J. Gao, B. Zhang, S. Cao, Y. Zhang, Z. Li, L. Sun, J. Hou, *Nat. Commun.* **2020**, *11*, 5462.

[46] Y. Wu, X. Tao, Y. Qing, H. Xu, F. Yang, S. Luo, C. Tian, M. Liu, X. Lu, *Adv. Mater.* **2019**, *31*, 1900178.

[47] C. Zhu, Z. Yin, W. Lai, Y. Sun, L. Liu, X. Zhang, Y. Chen, S.-L. Chou, *Adv. Energy Mater.* **2018**, *8*, 1802327.

[48] G. F. Chen, T. Y. Ma, Z. Q. Liu, N. Li, Y. Z. Su, K. Davey, S. Z. Qiao, *Adv. Funct. Mater.* **2016**, *26*, 3314-3323.

[49] L. Huang, D. Chen, G. Luo, Y. R. Lu, C. Chen, Y. Zou, C. L. Dong, Y. Li, S. Wang, *Adv. Mater.* **2019**, *31*, 1901439.

[50] M. Li, Y. Zhu, H. Wang, C. Wang, N. Pinna, X. Lu, *Adv. Energy Mater.* **2019**, *9*, 1803185.

[51] G. Chen, Z. Hu, Y. Zhu, B. Gu, Y. Zhong, H.-J. Lin, C.-T. Chen, W. Zhou, Z. Shao, *Adv. Mater.* **2018**, *30*, 1804333.

[52] M. Ledendecker, S. Krick Calderón, C. Papp, H.-P. Steinrück, M. Antonietti, M. Shalom, *Angew. Chem. Int. Ed.* **2015**, *54*, 12361-12365.

[53] L.-L. Feng, G. Yu, Y. Wu, G.-D. Li, H. Li, Y. Sun, T. Asefa, W. Chen, X. Zou, *J. Am. Chem. Soc.* **2015**, *137*, 14023-14026.

Author contributions

B.Z., S.L., C.S.Z., and C.C. conceived the idea and designed the project. B.Z. performed the major experiments and analyzed the results. Z.H.W., W.J.S., Y.G., W.W.W., T.M., and L.M. assisted with the figure production, data analysis, and experiment design. B.Z., S.L., and C.C. wrote and edited the manuscript. S.L., C.C., and C.S.Z. supervised the whole project. All authors discussed the results and commented on the manuscript.
